# Stress Hormone and Reproductive System in Response to Honey Supplementation Combined with Different Jumping Exercise Intensities in Female Rats

**DOI:** 10.1155/2014/123640

**Published:** 2014-02-09

**Authors:** Maryam Mosavat, Foong Kiew Ooi, Mahaneem Mohamed

**Affiliations:** ^1^Sport Science Unit, School of Medical Sciences, Universiti Sains Malaysia, 16150 Kubang Kerian, Kelantan, Malaysia; ^2^Department of Physiology, School of Medical Sciences, Universiti Sains Malaysia, 16150 Kubang Kerian, Kelantan, Malaysia

## Abstract

This study was performed to determine the effects of 8-week honey supplementation combined with different jumping exercise intensities on serum cortisol, progesterone, estradiol, and reproductive organs. Eighty-four 9-week-old female rats were divided into 7 groups: baseline controls (C_0_), sedentary group (C), 20 and 80 jumps per day (Ex_20J_, Ex_80J_), honey (H), and combined honey with 20 and 80 jumps per day (HEx_20J_, HEx_80J_) groups. Jumping exercise was performed at 5 days/week and honey was given at a dosage of 1 g/kg body weight/day for 7 days/week. The level of serum cortisol was higher in Ex_20J_ and Ex_80J_ compared to C. There was significantly lower value of serum cortisol in HEx_20J_ compared to Ex_80J_. Serum progesterone levels were significantly lower in Ex_20J_ and Ex_80J_ compared to C. However, serum progesterone levels were significantly higher in HEx_20J_ and HEx_80J_ compared to Ex_20J_ and Ex_80J_. Relative uterine weights were significantly greater in HEx_20J_ compared to C and HEx_80J_, respectively. There was no significant difference in estradiol level and relative ovarian weights among all the groups. Therefore, honey elicited beneficial effects in reducing the increase of cortisol and in increasing the reduce of progesterone levels induced by different intensities jumping exercise in female rats.

## 1. Introduction

Despite considerable health benefits provided by physical activity and exercise training, several studies have reported that female engaged in high volume of physical training may face the problems of reproductive disorders and osteoporosis [1–4]. It has been shown that prolonged strenuous physical activity is accompanied with disrupting in hypothalamus- pituitary ovarian (HPO) axis and resulting in disturbance of gonadotropin releasing hormone (GnRH) pulsatility. This GnRH disturbance is accompanied with an increase in follicular phase length and a decrease in luteal phase secretion in animal [5] and human [6]. High level of serum cortisol due to physiological stress induced by exercise can be a probable factor in suppression of gonadotropin pulsatility in female athletes [7, 8]. Low energy availability which is described as dietary energy intake minus exercise energy expenditure is also an important factor in resulting disruption in HPO and hypoestrogenism in female athletes [3, 9]. A longitudinal study done by Williams et al. (2001) [10] provided strong evidence on the role of low energy availability in the development of exercise-induced amenorrhea in monkeys.

Several previous studies reported that high volume of exercise depending upon intensity and duration may elevate reactive oxygen species (ROS) production produced by metabolic and physiological processes that can cause cellular damage such as lipid damage, inhibition of protein synthesis, and depletion of ATP [11–13]. Additionally, it was reported that there were physiological and pathogenesis effects of ROS on reproductive system [14]. A recent study showed that 8 weeks of intensive cycling training significantly increased the seminal plasma cytokines, seminal oxidative stress biomarkers of ROS and malondialdehyde (MDA), and decreased the levels of seminal antioxidants superoxide dismutase (SOD), catalase, total antioxidant capacity (TAC), and semen parameters in male cyclists [15]. Antioxidant enzymes such as catalase, superoxide dismutase, and glutathione peroxidase, and numerous nonenzymatic antioxidants, including vitamins A, E, and C, glutathione, and flavonoids, are free-radical scavengers which can decrease the formation and neutralize free radicals [14, 16, 17]. It has also been shown that ascorbic acid supplementation as a non-enzymatic antioxidant could elicit beneficial effects in improving steroid genesis in patients with luteal phase deficit history [17].

Honey as a natural complex of sugars has been used traditionally for its therapeutic effects. Honey contains carbohydrates such as glucose, fructose, sucrose and raffinose, enzymes, flavonoids, antioxidants, minerals, organic acids, proteins, phenolic acids, and vitamins such as vitamin C and vitamin E [18]. Tualang honey is a wild multiflora honey from Malaysian rain forest with intermediate Glycemic Index (65 ± 7) [19] and contains phenolic compounds which has shown good antioxidant activity [20]. In a study on cyclists, honey supplementation has showed significantly less elevation in seminal ROS and MDA as well as significantly less reduction in semen parameters following long-term intensive cycling training in male. The results of this study showed that in response to honey supplementation, cyclists' seminal plasma SOD, catalase, and TAC increased significantly after exercise compared with baseline values. The authors postulated that it may be possible that honey supplementation would be effective in reducing seminal plasma cytokines and oxidative stress biomarkers as well as increasing seminal antioxidant levels and probably would be related to improvements in spermatogenesis and fertility capacity in humans [15]. In a previous animal study done by Zaid et al. [21], administration of Tualang honey elicited beneficial effects in enhancement of uterine weight and thickness of vaginal epithelium in ovariectomised rats. Additionally, it has been shown by recent studies that that honey could elicit protective effect against cigarette smoke-induced impaired testicular functions and low testosterone level in rats partly through its antioxidant properties [22, 23].

However, to date no study has been carried out to investigate the benefits of honey to protect against adverse effects on stress hormone level and female reproductive system induced by high volume of physical activity. Moreover, we hypothesized that honey as a source of energy with antioxidant properties may have a protective role against adverse effects induced by exercise on stress hormone and female reproductive system. Therefore, the aim of this study was to investigate the effects of honey supplementation combined with different jumping exercise intensities, that is, low and high intensities on stress hormone, reproductive hormones, and reproductive organ weights in rats.

### 1.1. Animals

Eighty-four 9-week-old female Sprague-Dawley rats with mean initial body weight of 191.3 ± 13.4 g were obtained from the Animal Research and Service Center, Universiti Sains Malaysia (USM). The experimental protocol was approved by Animal Ethics Committee, USM (USM/Animal Ethics Approval 2011/[71][325]). 

### 1.2. Animal Grouping

The rats were divided randomly into 7 experimental groups with 12 rats in each group: baseline control group with rats which were sacrificed at the age of 9 weeks old at the beginning of the study (C_0_), sedentary group without honey supplementation and exercise (free cage activity) for 8 weeks (C), low intensity jumping exercise with 20 jumps per day at 5 days per week for 8 weeks (Ex_20J_), high intensity jumping exercise with 80 jumps per day for 5 days per week for 8 weeks (Ex_80J_), honey supplementation for 7 days per week for 8 weeks (H), low intensity of 20 jumps per day for 5 days per week combined with honey supplementation for 8 weeks (HEx_20J_), and high intensity of 80 jumps per day for 5 days per week combined with honey supplementation for 8 weeks (HEx_80J_).

### 1.3. Vaginal Smear and Body Weight Measurement

At the beginning and end of this study, vaginal smear was performed to confirm the diestrus phase of the rats for hormonal phase standardization. Vaginal secretion of each rat was collected with plastic pipette filled with 10 *μ*L of normal saline (NaCl 0.9%) by inserting the pipette tip into the rat's vagina, and then the unstained materials were observed under a light microscope at 100x magnifications. Initial and final body weights were measured to the nearest 0.1 g by using a body weight weighing scale (Navigator, OHAUS Corporation, N2B110, Switzerland).

### 1.4. Honey Supplementation

Tualang honey supplementation was given to the rats with a dosage of 1 g per kg body weight per day by oral gavage for 7 days per week. The rats in combined honey with jumping exercise groups (HEx_20J_ and HEx_80J_) were fed with honey, 30 minutes prior to the jumping exercise session. The dosage of honey was calculated based on the rat biweekly body weight [24].

### 1.5. Training Programme

The jump training protocol of this study was based on a previous study [24], with 5 days per week, that consisted of either 20 or 80 jumps per day for 8 weeks. Briefly, each rat in the jumping exercise groups was placed at the bottom of a specially designed wooden box, measuring 30.5 cm × 30.5 cm and 40 cm in length, width, and height, respectively. The jumping exercise was initiated by applying an electrical current to the wired floor (electrical grid) of the box through a stimulator. When stimulated, each rat jumped from the floor of the box to catch the top edge of the box with its forepaws. The rat was then immediately returned to the floor of the box to repeat the procedure. The time required per jump was about 4 seconds. After a few days of training, the rats jumped without electrical stimulation. Rats in the sedentary groups were also handled during the duration of the study without any electrical stimulus to mimic the stress induced by handling.

### 1.6. Blood and Reproductive Organs Collection and Measurements

After 8 weeks of experimental period, the rats were anaesthetised by lying for 2-3 minutes in a dried jar containing chloroform-soaked gauze pad; then, they were decapitated by using small guillotine (Scientific Research Instrument, UK). Blood from the decapitated site was collected into the 10 mL test tube through a funnel, allowed to completely clot and subsequently centrifuged at 4°C for 15 minutes at 4000 RPM (Health-Ratina 46RS, Germany). Serum was collected and stored at −80°C (Heto Ultra Freezer 3410, Denmark) for subsequent analysis of serum hormones. Levels of serum estradiol, progesterone, and cortisol were measured using commercial immunoassay kits (Rat estradiol ELISA kit, Cusbio, USA; rat progesterone ELISA kit, Cusabio, USA; rat cortisol ELISA kit, Cusbio, USA).

The reproductive organs, that is, uterine horns and ovaries, were carefully removed and cleaned and weighed by using an electronic balance (Denver Instrument Company, USA). Relative ovarian and uterine weights were obtained by dividing the absolute ovarian and uterine weights with final body weight, respectively.

### 1.7. Statistical Analysis

Statistical analysis was performed using Statistical Package of Social Sciences (SPSS) Version 18.0. All of the data are presented as mean ± standard deviation (SD). After checking normality and homogeneity, the data with normal distribution and homogenous variances were analysed using one-way analysis of variance (ANOVA) with post hoc (least significant difference) test to determine the significance of the difference between groups. *P* value of < 0.05 was considered as statistically significant and used for all the comparisons.

## 2. Results

Mean serum cortisol, estradiol, and progesterone levels are presented in Table 1. Serum cortisol levels were significantly (*P* < 0.05) lower in C, H, HEx_20J_, and HEx_80J_ compared to C_0_. There were significantly (*P* < 0.05) higher values of serum cortisol in Ex_20J_ and Ex_80J_ compared to C. The level of cortisol level was lower (*P* < 0.05) in HEx_20J_ compared to Ex_80J_. However, the cortisol levels in both HEx_20J_ and HEx_80J_ were not significantly different from C. There were no significant differences in estradiol level among all the groups. In serum progesterone, there were no significant difference in this measured parameter in all the groups compared to C_0_. However, serum progesterone levels were significantly (*P* < 0.05) lower in Ex_20J_ and Ex_80J_ compared to C. Meanwhile, serum progesterone levels were significantly (*P* < 0.05) higher in HEx_20J_ and HEx_80J_ compared to Ex_20J_ and Ex_80J_, respectively.

The means of final body weights, relative ovarian, and uterine weights of all the experimental groups are presented in Table 2. There were no significant differences in mean final body weight among all the groups. It was found that the relative ovarian weights were significantly greater (*P* < 0.05) in all the groups compared to C_0_. However, there was no significant difference in relative ovarian weights between C and all the other experimental groups. Relative uterine weights were significantly (*P* < 0.05) greater in HEx_20J_ compared to C and HEx_80J_, respectively_._

## 3. Discussion

In the present study, we observed that serum cortisol levels, as an indicator of physiological stress, were significantly higher in 20 and 80 jumps per day groups compared to sedentary group. This higher value of serum cortisol was accompanied with reduction in serum progesterone levels in jumping exercise groups that consisted of 20 and 80 jumps per day. The reason of the reduction in progesterone level in the exercise alone groups may be due to physiological stress induced by exercise training and low energy availability without honey supplementation in the rats.

It has been reported that exercise training can influence the activation of hypothalamic pituitary adrenal (HPA) axis and consequently increment of serum cortisol level in animal [25]. Similarly, increased cortisol level was observed in the exercised alone rats in the present study. Elevation in cortisol level can suppress the hypothalamic-pituitary-ovarian (HPO) axis and acts as a deleterious factor on luteinizing hormone (LH) pulsatility [26–29]. Consistently, reduced level of progesterone concentration in the exercised alone rats was found in the present study.

Regarding low energy availability, it was indicated by several previous studies that low energy availability but not stress of exercise may also alter LH pulsatility in exercising women [9, 10, 30]. It was reported that low energy availability by reducing the circulation of insulin [31] and insulin-like growth factor I (IGF-I) can cause increment of IGF-binding protein (IGFBP-1) level and cortisol level. This reduction in IGF-I activity can affect the stimulation of hypothalamic-pituitary-gonadal axis (HPG), whereas high level of cortisol concentration can inhibit hypothalamic secretion of GnRH [7]. As can be seen in the present study, low levels of serum progesterone were observed in the jumping rats without honey supplementation. Therefore, it is speculated that the scenario of low energy availability occurred in these rats.

The present study found that the level of serum cortisol in the rats in group of honey supplementation combined with 20 jumps per day was significantly lower compared to 80 jumps per day group without honey supplementation. It has been reported that Tualang honey has the highest source of phenolic acids and flavonoid compounds among Malaysian honeys which have strong free-radical-scavenging activities [32]. Similarly, another previous study has shown that honey supplementation can reduce oxidative stress and testicular damage in male rats exposed to cigarette smoke [22]. Thus it is speculated that honey supplementation may have played its role in reducing oxidative stress induced by jumping exercise.

In a study done by Williams et al. (2001) [10], it was reported that food supplement of about 138–181% of calorie intake that consisted of granola bars, dried fruit, fresh fruit, and commercial monkey chow could increase estradiol and progesterone levels with recovery times for circulating gonadotropin levels and reestablished ovulatory cycles during strenuous daily training in female amenorrheic monkeys. Their study indicated that low energy availability plays a causal role in the development of exercise-induced amenorrhea and reproductive dysfunction. In the present study, it was found that the values of serum progesterone were significantly higher in honey supplementation combined with 20 and 80 jumps per day compared to low and high intensity jumping exercise alone. This increment in progesterone levels could show that honey supplementation through its carbohydrates components such as fructose, glucose, maltose and sucrose [18] which equivalent to 313 calories per 100 g [21] and its antioxidant properties such as phenolic and flavonoid compounds [32, 33] may elicit protective effects against negative energy balance and oxidative stress induced by jumping exercise on both level of intensities. Therefore, it is speculated that the antioxidant properties of honey might be one of the reasons in protecting the altered serum cortisol and progesterone levels induced by jumping exercise in rats.

Low level of serum estradiol was reported by a previous investigation in female rats treated with honey supplementation at dosage of 0.2, 1, and 2 g/kg [21]. The present study showed slightly lower estradiol level among exercise groups and honey supplementation group compared to sedentary group even though the differences were not statistically significant. In a previous study, it was shown that intensive exercise without food restriction was not responsible for significant changes in estradiol concentration level [34]. The discrepancy between the present finding and previous studies may be due to differences in the experimental design.

The present study found that administration of honey supplementation at dosage of 1 g/kg body weight combined with 20 jumps per day (HEx_20J_) at duration of 8 weeks could increase relative uterine weight in female rats compared to sedentary rats and the rats fed by same dosage honey supplementation combined with 80 jumps per day. This present finding was consistent with previous investigation done by Zaid et al. (2010) [21] which found that Tualang honey increased uterine weight and thickness of vaginal epithelium in ovariectomised rats. These positive effects of honey may be due to its phenolic compound and flavonoids content [32], with the evidence of previous studies which reported the estrogenic activity of flavonoids particularly kaempferol and quercetin [35, 36]. Hence it is speculated that the increased uterine weight found in this study might be due to the estrogenic effect of phenolic compounds presence in honey. Additionally, it found that low jumping intensity of 20 jumps per day could increase uterine weights; however, intensity of exercise with 80 jumps per day did not show the same effect. The present study also showed that training protocol that consisted of 20 jumps and 80 jumps per day did not affect the ovaries weights in the exercised rats. Similarly, 1 g/kg body weight honey supplementation also did not affect relative ovary weights. The precise mechanisms of these observations are unclear and further study on the histological changes in the rats' ovary and uterus is warranted to confirm these observations. The limitations of the present study were that corticosterone which is a stress indicator besides cortisol, luteinizing hormone which is a reproductive hormone, and oxidative stress markers were not measured, and it is suggested to include these measured parameters in further studies.

## 4. Conclusion

Honey supplementation elicited effective role in increase of uterine weight in female rats. However, administration of Tualang honey elicits beneficial effects to diminish the adverse effects induced by jumping exercise on female reproductive hormones and stress hormone in rats. Therefore, honey supplementation may be beneficial for female athletes for maintaining their normal reproductive functions. Nevertheless, further human study is needed to confirm the present study.

## Figures and Tables

**Table 1:** Serum cortisol, serum progesterone, and serum estradiol concentrations (mean ± SD).

Group	Cortisol	Progesterone	Estradiol
	pg/mL	ng/mL	pg/mL
C_0_	1.86 ± 0.31	120.30 ± 128.3	22.88 ± 7.00
C	1.20 ± 0.40^a^	135.75 ± 49.8	33.44 ± 11.52
H	1.32 ± 0.33^a^	114.34 ± 120.6	31.93 ± 6.97
Ex_20J_	1.65 ± 0.59^b^	76.24 ± 29.1^b^	30.60 ± 10.67
Ex_80J_	1.63 ± 0.31^b^	62.19 ± 44.2^b^	26.07 ± 8.84
HEx_20J_	1.19 ± 0.44^a,d,e^	191.05 ± 153.2^d,e^	28.70 ± 12.60
HEx_80J_	1.35 ± 0.56^a^	146.85 ± 85.5^d,e^	31.06 ± 6.73

Data were analysed by one-way ANOVA.

^a^Significantly different from C_0_ (*P* < 0.05), ^b^significantly different from C (*P* < 0.05), ^c^significantly different from H (*P* < 0.05), ^d^significantly different from Ex_20J_ (*P* < 0.05), and ^e^significantly different from Ex_80J_ (*P* < 0.05).

C_0_: baseline control; C: sedentary without honey and jumping exercise; H: honey supplementation of 1 g/kg body weight/day; Ex_20J_: low intensity of 20 jumps/day; Ex_80J_: high intensity of 80 jumps/day; HEx_20J_: combined honey supplementation with low intensity of 20 jumps/day; HEx_80J_: combined honey supplementation with high intensity of 80 jumps/day.

**Table 2:** Final body weights, relative uterine, and ovarian weights (mean ± SD).

Groups	Final body weights (g)	Relative uterine weight (mg/kg body weight)	Relative ovarian weight (mg/kg body weight)
C_0_	196.5 ± 17.1	1.53 ± 0.61	0.57 ± 0.09
C	245.6 ± 19.1	1.71 ± 0.56	0.41 ± 0.08^a^
H	251.9 ± 19.8	1.95 ± 0.27	0.40 ± 0.10^a^
Ex_20J_	250.6 ± 11.8	1.90 ± 0.40	0.40 ± 0.11^a^
Ex_80J_	250.3 ± 18.0	1.90 ± 0.30	0.45 ± 0.10^a^
HEx_20J_	246.7 ± 22.4	2.26 ± 0.11^a,b^	0.40 ± 0.07^a^
HEx_80J_	254.6 ± 20.6	1.77 ± 0.27^c^	0.39 ± 0.06^a^

Data were analysed by one-way ANOVA.

^a^Significantly different from C_0_ (*P* < 0.05).

^b^Significantly different from C (*P* < 0.05).

^c^Significantly different from HEx_20J_ (*P* < 0.05).
